# Performance Study of Portable Semiconductor Refrigeration Device Based on CFD Simulation

**DOI:** 10.3390/mi14020296

**Published:** 2023-01-23

**Authors:** Bin Li, Feng Wang, Feng Jiang, Shaocong Zhao, Shutao Wei, Piaolin Peng, Xiangdong Wang, Anna Jiang

**Affiliations:** 1 Institute of Manufacturing Engineering, Huaqiao University, Xiamen 361021, China; 2 College of New Materials and Footwear Engineering, Liming Vocational University, Quanzhou 362000, China; 3 P. E. Department, Xiamen University of Technology, Xiamen 361000, China; 4 Innovation and Research Center, 361° (CHINA) Co., Ltd., Xiamen 361000, China; 5 School of Physical Education, Jimei University, Xiamen 361021, China; 6 College of Mechanical Engineering and Automation, Huaqiao University, Xiamen 361021, China

**Keywords:** computational fluid dynamics simulation, portable cooling device, semiconductor refrigeration technology

## Abstract

Since the summer of 2022, the whole world has suffered the abnormal weather phenomena of high ambient temperature. Equipment for refrigeration, particularly portable refrigeration equipment, is crucial for personal protection in high–temperature environments, but cooling performance and miniaturization have been challenging issues. A portable air conditioner based on a semiconductor refrigeration device for human body cooling was developed. The total weight of the device is 450 g. The overall power consumption of the device is 82 W and the energy consumption ratio of semiconductor cooling plate is 0.85. The semiconductor refrigeration technology is based on the Peltier effect, supplemented by a DC fan to send the cooling air out to a specified position or zone. The structural parts are manufactured by 3D printing technology to make the overall size of the device more compact. The air volume and cooling performance of the device were analyzed by computational fluid dynamics simulation and the temperature distribution was measured by an infrared thermal imager and other instruments, and the measured results agreed with the CFD simulation results. The test ambient temperature was 20 °C. The measurement results showed that the wind speed of the hot air outlet was 6.92 m/s and that of the cold air outlet was 8.24 m/s. The cold air surface temperature reached a stable state of 13.9 °C in about 4 min, while the hot air surface temperature reached a stable state of 47.2 °C.

## 1. Introduction

The global climate is warming, making the summer months begin to experience frequent extreme heat. On average, the global population experienced three times as many extreme heat days in 2020 than it did in 2008. Extreme heat has been relentless across the Northern Hemisphere this summer and threatens people’s lives. In addition, the current repeated prevention and control measures related to the novel coronavirus pneumonia epidemic tend to be normal. A large number of medical workers wear protective clothing to participate in the work. The high–temperature environment in clothing endangers the health of medical staff. At present, there have been studies on the detection and monitoring of human physiological signals by wearable electronic devices [1,2,3,4], but even more important is cooling the body when it becomes too hot. Therefore, workers operating in high–temperature environments are in urgent need of a portable cooling device combined with special clothing (such as welding suits, medical protective clothing, etc.). Taking epidemic prevention front–line medical staff as an example, there are traditional cooling methods such as turning on fans, turning on air conditioners in isolation rooms, and touching ice cubes. There are also new clothes with positive pressure protective features for cooling down. However, the above cooling methods have problems such as low refrigeration efficiency, high energy consumption, and poor portability. In addition, for the large temperature difference between day and night in the field, there are still few studies on portable refrigeration and heating two–way–control equipment. Semiconductor refrigeration produces heat while cooling. Taking advantage of this feature, the portable device can be used in hot conditions during the day and can be used at night for heating inside a field tent. Therefore, this paper uses semiconductor refrigeration technology to design and build a small and low–power consumption portable refrigeration device, and study the performance of the device based on CFD simulation.

**Figure 1:**
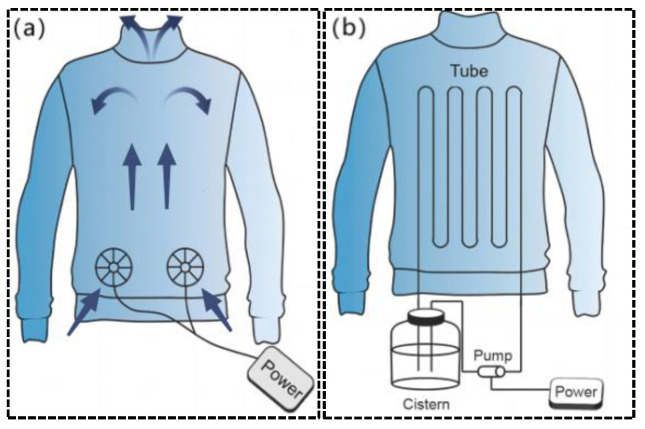
Schematic diagram of gas and liquid heat transfer. (**a**) Air cooling suit; (**b**) liquid cooling suit.

Current research on portable cooling equipment is focused on the “microclimate zone” within clothing [5], which is a thin layer of air between the interior of the clothing and the surface of the body skin. The main factors affecting physical comfort in this zone include the flow rate, temperature, and humidity of the fluid in the zone [6]. The adjustability of the cooling performance, such as the adjustment of air velocity, flow rate, or temperature, should be considered in the device design. Gas and liquid heat transfer are the most common cooling methods, as shown in Figure 1. The principle of gas heat transfer is to use a fan to blow the gas into the clothing and discharge it from the opening of the clothing so as to accelerate the evaporation of sweat on the surface of the body and achieve cooling effect, while the principle of liquid heat transfer is to use low–temperature liquid to take away the heat on the contact surface to achieve the cooling effect. They both convert the external electrical energy into the kinetic energy of the fluid medium [7,8]. The rapid flow of the fluid accelerates the heat transfer process, which is used to control the temperature distribution in the “microclimate zone”. The limitation of the above two methods is that they can only achieve separate refrigeration, and the size and weight of the liquid cooling equipment is greater.

**Figure 2:**
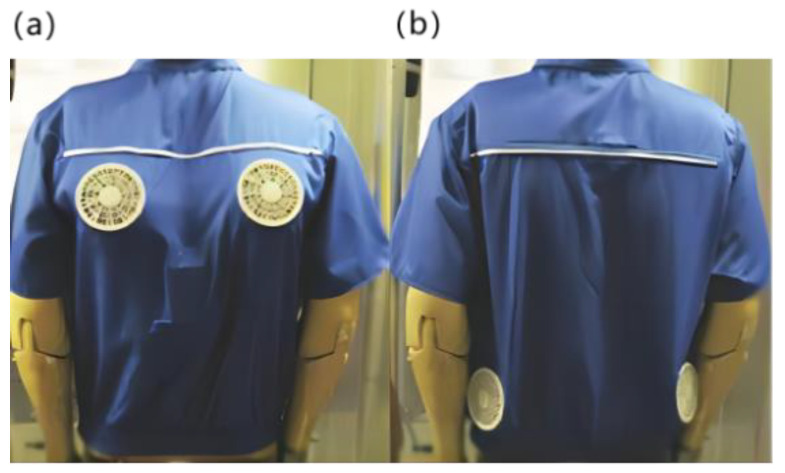
Ventilation jacket [9]. (**a**) The fan was set on the back. (**b**) The fan was set at the waist.

In the study of gas cooling, Zhao et al. [9] produced an air–cooled suit. A miniature fan and a battery were integrated into the garment, and the miniature fan enhanced forced convection to increase the evaporation rate of the sweat. It can accelerate the temperature reduction of the body surface and microclimate zone. Zhao et al. [9] made five jackets with the same fan airflow size and only the installation position was located in different positions of the garment with closable openings in order to study the cooling effect of different fan positions, as shown in Figure 2. The same battery and micro–fan are integrated in all jackets. Each ventilation unit was a small fan with a diameter of 10 cm. They can circulate ambient air around the trunk at a flow rate of approximately 0.012 m/s. The test results show that the fan at different locations has a more pronounced local impact, but only with the cooling function, and the cooling rate is slow and takes about 15 min to reach a steady state.

**Figure 3:**
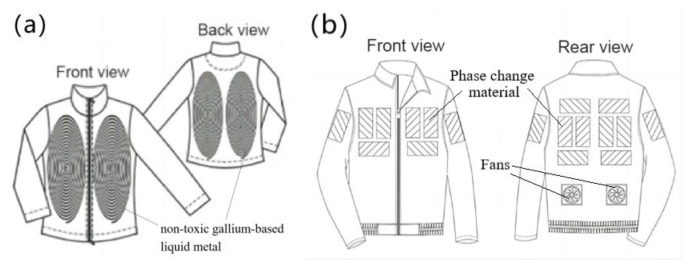
Clothing with integrated refrigeration components. (**a**) Liquid metal–based active cooling suit [11]. (**b**) Personal cooling suits combining ventilation systems and phase change materials [12].

For the study of traditional water–cooled clothing, Guo et al. [10] conducted relevant research on liquid cooling clothing. The theoretical model of heat transfer from the human skin surface to the environment shows that the maximum working duration of liquid–cooled clothing is 3.36 h at an ambient temperature of 45 °C, a flow rate of 224.5 mL/min, and a maximum cooling rate of 243.2 W/m^2^, and the experimental results were within 10% error from the calculated results [10]. Although Guo et al. have balanced the cooling effect and weight of the liquid cooling suit, its weight still reached 2 kg. At the same time, the cooling effect decreases with the increase of liquid temperature. Rahman et al. [11] creatively used a non–toxic gallium–based liquid metal [12] as the cooling medium for the liquid cooling suit system, which makes it possible to carry more heat in a smaller volume, as shown in Figure 3. This active cooling system is one–third lighter than that of conventional liquid cooling systems, while the cooling effect is four times longer than that of conventional systems [6]. However, it still requires liquid storage devices, and there is a risk of liquid leakage. In addition, Lu et al. [13] and Kang et al. [14] combined the function of ventilation and heat dissipation to develop a personal lightweight cooling system with better results, as shown in Figure 3. The system uses phase change material for heat dissipation. Its power consumption is greater than that of air cooling and liquid cooling. Meanwhile, the continuity of phase change refrigeration is not good. It can only provide a cooling time of 90 to 110 min in hot and humid environments and 20 to 30 min in cold and dry environments. Due to the degradation of thermal physical properties during the use of phase change materials, its durability needs to be further studied.

**Figure 4:**
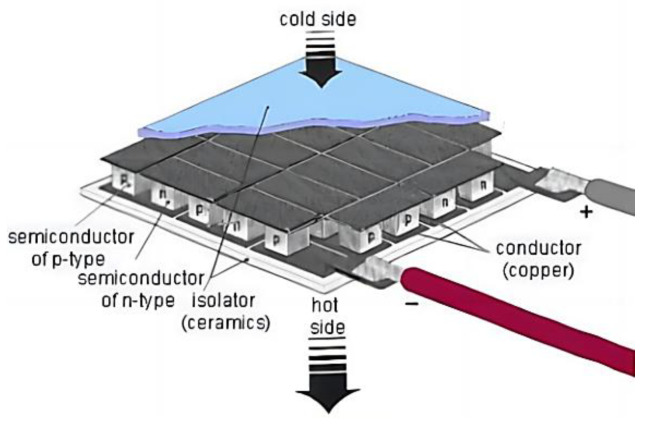
Schematic diagram of semiconductor refrigeration technology.

In terms of heating in microclimates, flexible heaters based on stretchable polymers with conductive compositions have seen a number of advances. Current studies in this direction include a metal nano–network [15], copper–plated fibers [16], and graphite [17]. However, these flexible heaters tend to increase local resistance during deformation, resulting in local high temperatures. The core component of the portable refrigeration device designed in this paper is a commercial semiconductor cooling plate, which can greatly reduce the cost of making the refrigeration device. The semiconductor refrigeration method involved can be classified as a heat pump in the usual sense, and it is also commonly referred to as a thermoelectric refrigeration chip, as shown in Figure 4. The main principle is the use of some semiconductor materials that have the Peltier effect to achieve the purpose of cooling, and it is a reversible process. It can convert heat into electricity. Zhang et al. [18] reviewed the research progress and proposed the research direction. For refrigeration–related applications, Alwan et al. [19] installed a cooling chamber with thermoelectric elements on top of the solar still to enhance condensation. Miao et al. [20] also designed a new type of plate–type ground source radiation system for space heating and cooling in buildings by utilizing the characteristics of semiconductor refrigeration technology. Compared to other refrigeration components, it has the advantage of high reliability and small size and does not require high space size. It needs timely and effective heat dissipation of its heating surface, and this heat can also be used to heat microclimates when heating functions are needed. The semiconductor refrigeration technology has been applied to target cooling devices, refrigeration storage cabinets, lithium battery temperature control devices, and other fields. Semiconductor refrigeration technology in which a semiconductor cooling plate directly wraps around the heat dissipation surface is used for thermal conductivity cooling. It can easily reach sub–zero temperatures in a short period, and the choice of the power of the semiconductor cooling plate makes the time to reach the maximum temperature difference different. The device behaves similarly to phase change refrigeration in terms of overall power consumption, but the lowest temperature it can reach with the same power is difficult to achieve with other refrigeration principles. It meets the conditions of use in the application of a portable semiconductor refrigeration device and facilitates miniaturized design.

**Figure 5:**
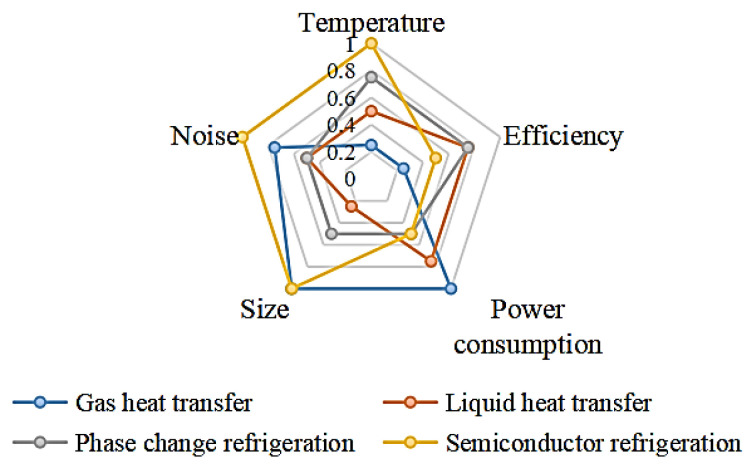
Comparison of different refrigeration methods.

The purpose of this paper is to develop a portable semiconductor refrigeration device. It can also realize the functions of refrigeration and heating at the same time and has a very wide application prospect. The comparative analysis of the five aspects of large temperature difference, high efficiency, low energy consumption, miniaturization, and low noise is shown in Figure 5. Based on the above situation, semiconductor refrigeration technology can overcome the limitations of existing technologies applied to portable refrigeration devices after considering miniaturization and an excellent refrigeration effect under the same power consumption. In this paper, we use computer modeling and CFD simulation to assist in the design and build and test of the actual device based on the simulation results. Finally, we provide a small portable refrigeration device based on semiconductor refrigeration technology which is lightweight and can actively cool down the temperature, making it possible to regulate the temperature inside the garment more flexibly and actively.

**Figure 6:**
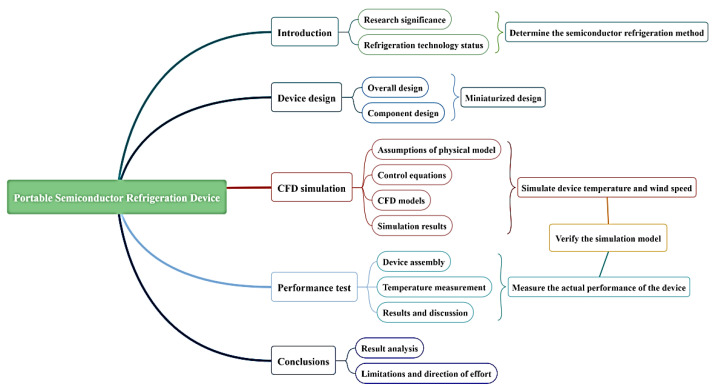
The workflow of portable refrigeration device in this paper.

In this paper, we use computer modeling and CFD simulation to assist in the design and build and test of the actual device based on the simulation results. Finally, we provide a small portable refrigeration device based on semiconductor refrigeration technology which is lightweight and can actively cool down the temperature, making it possible to regulate the temperature inside the garment more flexibly and actively. The workflow is shown in Figure 6.

## 2. Device Design

### 2.1. Overall Design

**Figure 7:**
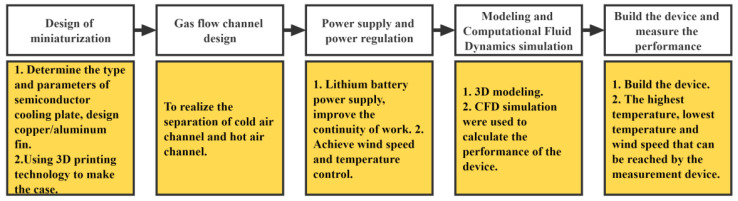
The overall design of the device.

**Figure 8:**
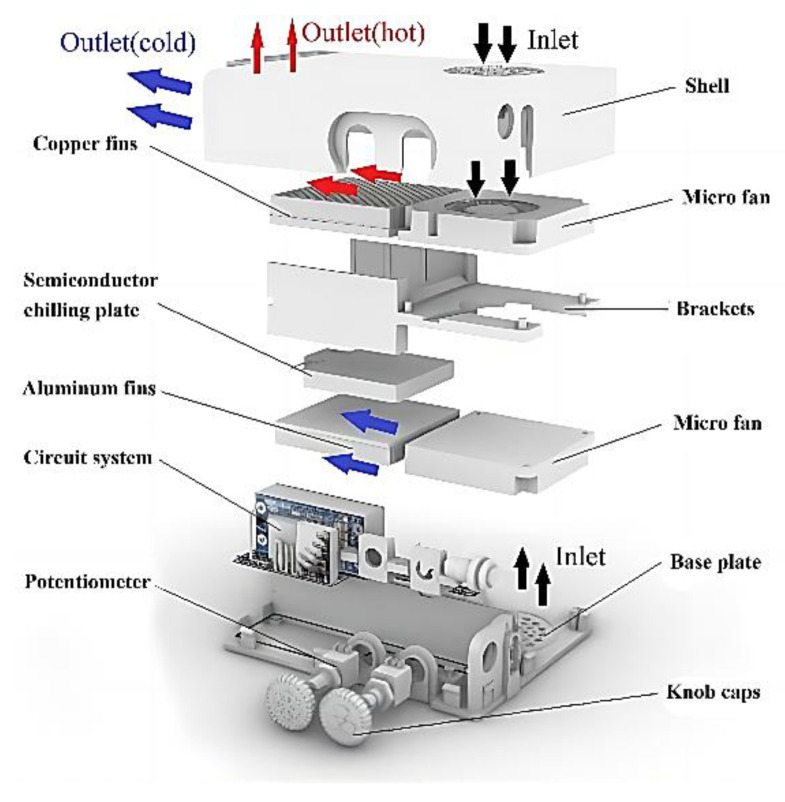
Exploded view of the portable refrigeration device.

To achieve the miniaturization design and low power consumption design requirements of the portable refrigeration device, which is based on a semiconductor cooling plate as the core component so that the device can achieve lower temperatures with the same power consumption as other refrigeration methods. To achieve a miniature design, a combination of air–cooling and the use of a semiconductor cooling plate for cooling is used. The overall design of the device is shown in Figure 7. The design program uses Solid Works software for 3D modeling. The software version used in this paper is SOLIDWORKS 2020, from Dassault Systemes, a European company. The main structure and flow field arrangement are shown in Figure 8.

The basic working principle is that after the semiconductor cooling plate is energized, both the hot side and the cooling side use thermal conductive silica gel to adhere to the fins to increase the thermal conductivity area. To provide good heat dissipation to the semiconductor cooling plate, copper fins with higher thermal conductivity are used for heat dissipation, and aluminum fins with lower density are used on the cooling side of the semiconductor cooling plate to reduce the overall weight of the device. Two miniature fans are arranged at the same end of the two fins, one to blow out the cold air and the other to dissipate heat to ensure the normal working of the refrigeration components. The two miniature fans are arranged with the air outlets blowing towards the fins in the same direction and the air inlets placed back to back so that the hot and cold air flow channels are separated. The cooling power and air speed of the device are controlled separately by adjusting the voltage. In order to reduce the cost of building the device, the above main components refer to the size of the existing components on the market for the structural design of the housing and other components.

### 2.2. Component Design

After the overall design scheme is determined, the core component of the portable refrigeration device is determined to be a semiconductor cooling plate. The common refrigeration equipment in life—air conditioning—is used as a reference. The lowest temperature of on traditional air conditioner is 16 °C, while the human comfort temperature is 26 °C. Therefore, to ensure the cooling performance of the device, the initial setting of the cold air outlet temperature is in the scope between 16 °C and 26 °C. To select the semiconductor cooling plate model with the appropriate parameters, the following is a calculation of the required cooling power.

These calculations refer to the relevant study by Zhao et al. [21]. In the steady state, the heat dissipation flux (*q*) from the body to the surrounding air can be expressed as Equation (1):

(1) $$q=\frac{T_{skin}-T_{amb}}{R_{cl}}$$

where *T_skin_* is the average skin surface temperature, *T_amb_* is the ambient air temperature, and *R_cl_* is the overall thermal resistance of the clothing. Although the metabolic rate of each individual is affected by various aspects, the value of ambient air temperature was set at 23.9 °C, when the metabolic power of the human body is 105 W [22]. This metabolic efficiency value is used as a reference to calculate the process of heat dissipation for the human body generally at rest.

According to the set performance of the portable cooling device to make the air around the human body down to a minimum of 16 °C, it is necessary to recalculate the heat dissipation flux (*q*′), that is, Formula (2).

(2) $$q^{\prime }=\frac{T_{skin}-T_{amb}^{\prime }}{R_{cl}}$$

where *T*′*_amb_* is the ambient air temperature for the design minimum temperature of 16 °C. For computational simplicity, it is assumed that the overall thermal resistance (*R_cl_*), such as clothing, is constant, while the human skin temperature (34 °C for the human body [23,24]) is considered to be constant.

The heat dissipation flux ratio in the natural state and the state with the participation of the semiconductor cooling plate can be obtained by combining Equations (1) and (2), i.e., Equation (3):

(3) $$\frac{q}{q^{\prime }}=\frac{T_{skin}-T_{amb}}{T_{skin}-T_{amb}^{\prime }}$$

According to the above equation, the heat dissipation flux *q*′ = 187 W with the participation of the semiconductor cooling plate, the difference between *q* and *q*′ is the additional heat dissipation flux required for the human body in the stationary state, i.e., *Q_e_* = 82 W.

To achieve these additional heat dissipation fluxes, the cooling power of the required semiconductor cooling plate needs to be further calculated on this basis. It is assumed that there is an adequate heat exchange between the skin and the air in the microclimate zone, while the skin surface is dry without considering the heat absorption by evaporation of liquid. Using Equation (4), the additional heat dissipation flux from the body can be calculated from the difference between the skin temperature and the device outlet temperature *Q_e_*:

(4) $$Q_{e}=c_{p}m_{c}\left(T_{skin}-T_{amb}^{\prime }\right)$$

where *c_p_* is the specific heat of the air and *m_c_* is the air mass flow rate. Further, the cooling power of the semiconductor cooling plate is calculated based on the difference between the inlet air temperature and the ambient temperature, as in Equation (5):

(5) $$Q_{c}=c_{p}m_{c}\left(T_{in}-T_{amb}^{\prime }\right)$$

where *T_in_* is the external ambient temperature, i.e., the temperature of the air inlet. To facilitate the subsequent test of the portable refrigeration device, the external environment was set to the ambient temperature of 20 °C at the time of the test. Equations (4) and (5) are compared to obtain Equation (6):

(6) $$Q_{c}=Q_{e}\frac{\left(T_{in}-T_{amb}^{\prime }\right)}{\left(T_{skin}-T_{amb}^{\prime }\right)}$$

This can be a rough calculation of the cooling power of the semiconductor cooling plate *Q_c_* = 41 W. Considering the various losses in the heat transfer process as well as the cost and reliability of the device, the semiconductor cooling plate selected is the XH–C1206 model, which is currently mainstream in the market. Its rated voltage is 12 V, the instantaneous starting current is 6 A, the cooling capacity is 61 W, and the energy consumption ratio is 0.85. Its dimension and the curve of temperature difference with time for this model of semiconductor cooling plate is shown in Table 1. The semiconductor cooling plate can obtain a stable temperature difference within 40 s.

Semiconductor cooling plates are prone to failure or even damage at high temperatures, so the heating surface uses thermally conductive silicone to adhere to the high thermal conductivity of the purple copper fins, while the cooling surface of the semiconductor cooling plate uses aluminum fins, which can increase the heat transfer area and minimize weight at the same time. To further dissipate the heat generated by the hot surface of the semiconductor cooling plate in time as well as the cooling surface of the cold air blowing out, here we reference the current market positive pressure garment air intake. The final choice was a micro–fan with a power consumption of 5 W and 4 cubic feet per minute. Accordingly, Formula (7) can be calculated according to the micro–fan outlet wind speed.

*q* = *vf* (7)

where *v* is the wind speed and *f* is the cross–sectional area of the air duct. Measurement of the cross–sectional area of the micro–fan outlet based on the above equation can be derived from the micro–fan outlet wind speed of 7 m/s. Manufacturers, countries, and specifications of major components are shown in Table 1.

## 3. Performance Analysis Based on CFD Simulation

### 3.1. Assumptions of Physical Model

**Figure 9:**
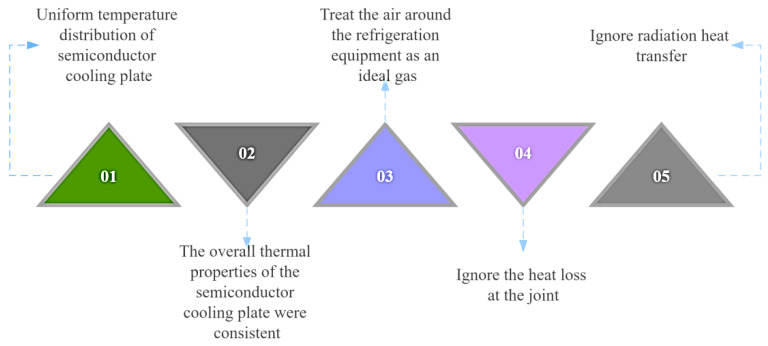
Model assumptions for CFD simulation.

Simulating the performance of semiconductor refrigeration devices easily leads to a large amount of calculations and even non–convergence of results when more conditions are considered. Therefore, before establishing the control equations, the temperature field distribution of portable refrigeration devices based on semiconductor refrigeration technology needs to be decomposed into several basic models, including heat conduction, convective heat transfer, and radiation heat transfer. In order to simulate the solution process as close as possible to the actual situation while reducing the computational effort and facilitating convergence, the model with a small amount of influence is simplified or ignored. The model assumptions in CFD simulation in this paper are shown in Figure 9.

### 3.2. Control Equations

The finite element analysis technique is an important means of modern engineering research [25] and improving the quality of human life [26]. Since the development of CFD technology, it has been widely used in the analysis of fluid flow, heat conduction, and other related physical phenomena. It is widely used in the comparative analysis of engineering field [27]. Nguyen, T. [28] selected the k–e turbulence model to study the effects of the straight fins and V–fins on the numerical simulation results of heat dissipation. The research showed that the effect of the fin shape on the heat transfer was not different when other conditions are equal. In terms of use for cooling, Jing–Ming Dong et al. [29] also used CFD to study a miniature steam ejector refrigeration system, which is embedded with a capillary pump loop that can result in a compact design.

The Fluent software version used in this paper is Ansys 2021 R2, which is a software application from ANSYS, Inc in the United States. Due to the fast air velocity inside the device, the state of the gas at the outlet of the micro–fan is not smooth, and the flow is in a non–stationary state. After checking the relevant cases, the turbulence model in Fluent software and the standard *k*−*ε* model with good generality can be used to solve the simulation of the gas flow in the semiconductor cooling device in this design, where the transport equations for the standard *k*−*ε* turbulence model are as follows in Equations (8) and (9).

(8) $$\frac{\partial \left(\rho k\right)}{\partial t}+\frac{\partial \left(\rho ku_{i}\right)}{\partial x_{i}}=\frac{\partial }{\partial x_{j}}\left[\left(\mu +\frac{\mu_{t}}{\sigma_{k}}\right)\frac{\partial k}{\partial x_{j}}\right]+G_{k}+G_{b}-\rho \varepsilon -Y_{M}+S_{k}$$

(9) $$\frac{\partial \left(\rho \varepsilon \right)}{\partial t}+\frac{\partial \left(\rho \varepsilon u_{i}\right)}{\partial x_{i}}=\frac{\partial }{\partial x_{j}}\left[\left(\mu +\frac{\mu_{t}}{\sigma_{\varepsilon }}\right)\frac{\partial k}{\partial x_{j}}\right]+C_{1\varepsilon }\frac{\varepsilon }{k}\left(G_{k}+C_{3\varepsilon }G_{b}\right)-C_{2\varepsilon }\rho \frac{\varepsilon^{2}}{k}+S_{\varepsilon }$$

where *G_k_* is the turbulent energy term due to the laminar velocity gradient; *G_b_* is the turbulent energy term due to buoyancy; *Y_M_* is the contribution term to the dissipation rate due to the expansion of the turbulent pulsations in the compressible flow to the global flow; *C*_1*ε*_, *C*_2*ε*_, *C*_3*ε*_ are three constants; *σ_k_* is the *k*–equation turbulence Prandtl number; *σ_ε_* is the *ε*–equation turbulence Prandtl number; *S_k_* is a user–defined turbulent energy term; *S_ε_* is a user–defined turbulence dissipation source term; *u_i_* is the velocity of *i*–phase flow at moment *t*; *x_i_* is the spatial position of the flow mass point in phase *i* at time *t*; *x_j_* is the spatial position of the flow mass point in phase *j* at time *t*; *μ* is the hydrodynamic viscosity; and *μ_t_* is the turbulent viscosity coefficient.

In this simulation calculation, *C*_1*ε*_ is 1.44, *C*_2*ε*_ is 1.92, *σ_k_* is 1.0, *σ_ε_* is 1.3, and the turbulent viscosity coefficient is calculated by Equation (10):

(10) $$\mu_{t}=\rho C_{\mu }\frac{k^{2}}{\varepsilon }$$

where *C_μ_* is the constant 0.09. The default values of the above parameters are those set in the FLUENT software.

### 3.3. CFD Models

The scientificity of model establishment determines the degree to which the simulation results reflect the real situation, and at the same time, the deviation of the simulation results can be controlled within a suitable range. Further, numerical calculations and performance simulations can be performed for the internal temperature field of portable refrigeration devices based on semiconductor refrigeration technology. The fluid and solid domains of the device are first modeled using Solid works software. Among them, the fluid domain includes cold air fluid and hot air fluid, and the solid domain includes a semiconductor cooling plate, copper fins, and aluminum fins and shell. Redundant parts that are not useful for the simulation process (circuit parts such as potentiometers) to reduce the meshing effort were removed. The total length of the model is 123.5 mm, the total width is 89 mm, the total height is 29.7 mm, and the volume is 125,887.34 mm^3^.

**Figure 10:**
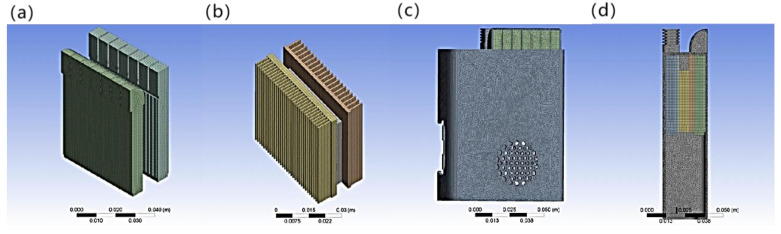
Finite element analysis meshing: (**a**) fluid domain; (**b**) solid domain; (**c**) solid domain and fluid domain as a whole; (**d**) solid domain and fluid domain cross–section.

The above modeled solid domain and fluid domain were meshed. In this meshing, adaptive sizing is used with a cell size of 0.001 m, a span angle center set to fine, the default grid average surface area of 9.9993 × 10^−5^ m^2^, and the minimum edge length of 1.1514 × 10^−6^ m. The fluid domain is divided into heat dissipating air fluid as well as cooling air fluid, and the mesh division results are shown in Figure 10a (left is heat dissipating air fluid, right is cooling air fluid). The mesh division results of aluminum fins, copper fins and semiconductor cooling plate are shown in Figure 10b, and the overall mesh situation and cross–section are shown in Figure 10c,d. The number of grid nodes is 236,325, the number of cells is 639,385, and the quality of the grid is good to meet the simulation requirements.

In order to verify the independence of different mesh densities, three mesh sizes are selected: 0.0005 m, 0.001 m, and 0.002 m. Because the meshing settings of hot surface simulation and cold surface simulation are the same, one simulation can be selected for verification. The hot surface simulation is taken as the verification object. The verification results are shown in Table 2. The results show that the variation of mesh density does not affect the accuracy of the final calculation results but also can predict the performance of the device. It can be concluded that the mesh density above is reasonable.

Each material was created in Fluent, including plastic shells, copper, and bismuth telluride (Bi_2_Te_3_), the substrate material for the semiconductor cooling plate, and material parameters were entered to match the material properties to each solid and fluid domain. In particular, this simulation involves energy exchange, so it is necessary to open the energy equation. The heat source is set for the semiconductor cooling plate, and the value of the energy source term is calculated based on the semiconductor power and refrigeration workload and the semiconductor refrigeration wafer size.

For the setting of the entrance boundary conditions, the velocity is set to a uniformly distributed constant. The wind speed in the *x*–axis direction is set to *v* = 7 m/s at the outlet of the micro–fan based on the calculation results. The *y*–axis and *z*–axis directions are ideal flow rates, both set to zero (where the *x*–axis direction is perpendicular to the end face of the micro–fan outlet and parallel to the cold/hot air flow path axis). The initial temperature is 20 °C and the exit boundary condition is kept as default for the free port.

### 3.4. Simulation Results

**Figure 11:**
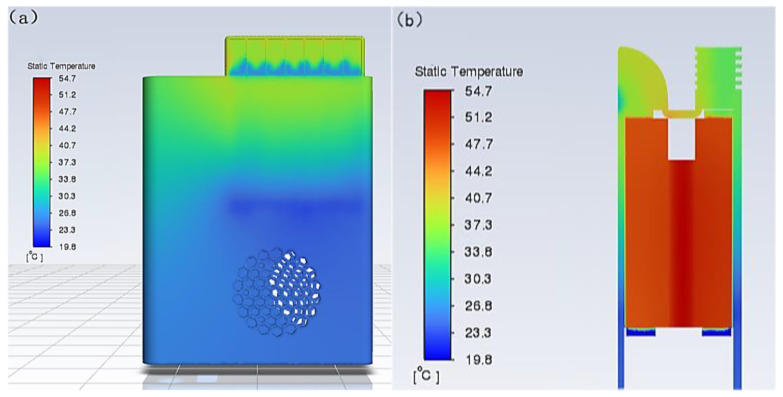

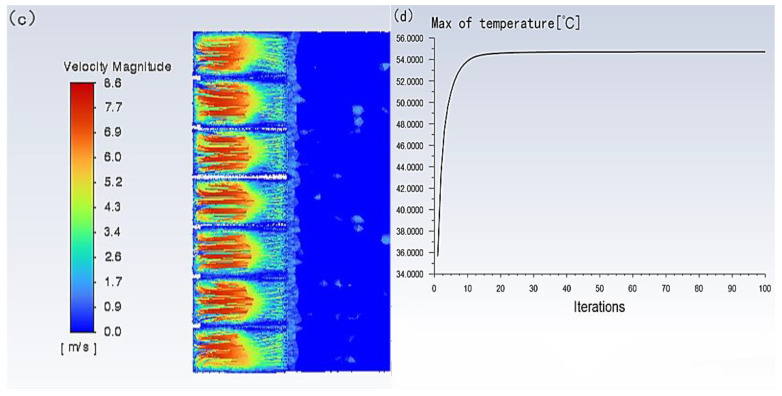
Simulation results of hot air surface. (**a**) Simulated temperature distribution cloud of the hot air surface of the portable refrigeration device. (**b**) Simulated temperature cloud of hot air section of portable refrigeration device. (**c**) Portable refrigeration device heat flow outlet velocity traces. (**d**) Maximum temperature variation curve of portable refrigeration device.

**Figure 12:**
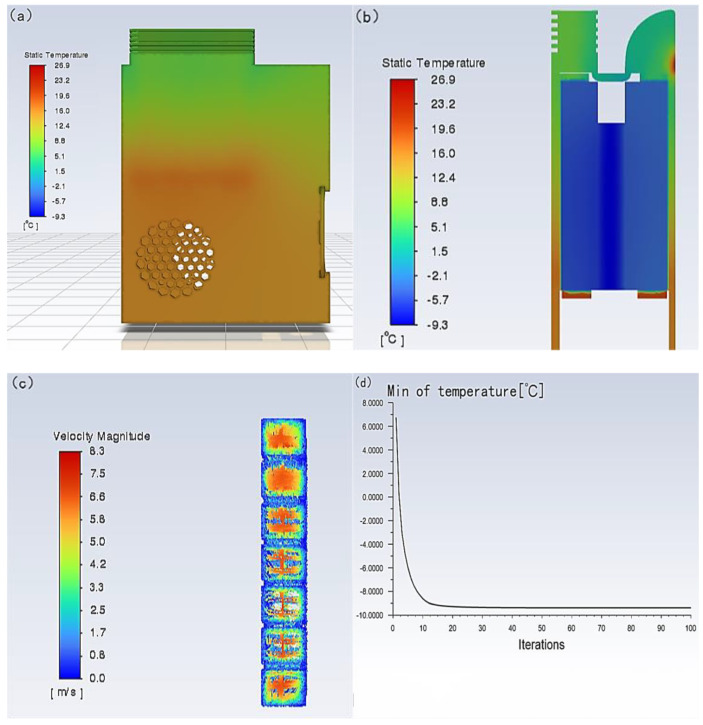
Simulation results of cold air surface. (**a**) Simulated temperature distribution cloud of the cold air surface of the portable refrigeration device. (**b**) Simulated temperature cloud of cold air section of portable refrigeration device. (**c**) Trace diagram of the cold flow outlet velocity of the portable refrigeration device. (**d**) Minimum temperature variation curve of portable refrigeration device.

The results of the solution are shown in Figure 11 and Figure 12 of the following series, including the overall temperature, section temperature, and outlet flow rate. It can be seen that the temperature of the hot air surface can reach 54.7 °C near the semiconductor cooling plate and about 40 °C at the exit, and the wind speed can also reach 8.6 m/s. The temperature of the cold air surface can reach −9.3 °C near the semiconductor cooling plate and about 8 °C at the exit, and the wind speed can reach 8.3 m/s.

The simulation results of the semiconductor cooling device can meet the performance requirements of air temperature and air speed compared to the design performance requirements. To a certain extent, it can show that the device is feasible and provides some guidance for building the device entity and realizing the designed performance requirements.

## 4. Performance Test Based on Experiments

### 4.1. Device Assembly

**Figure 13:**
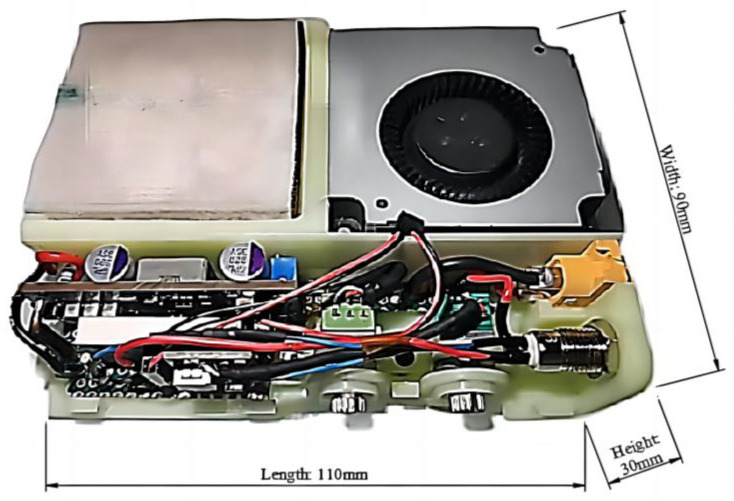
Physical diagram of the device (with the shell removed).

After the main structural parts and components of the portable refrigeration device are determined, the circuit is connected to achieve the function. Considering that the heat generated on the hot surface of the semiconductor cooling plate needs to be dissipated in time during use, the KSD–01F system’s touch–sensitive thermostat is installed on the copper fins of the hot side in order to avoid permanent damage caused by the high temperature of the component due to poor heat dissipation. The working principle of this model for the temperature control switch is the internal bimetal, which is temperature sensitive and is used to realize the conduction and disconnection of the contact points. Referring to the operating temperature range of semiconductor coolers, the KSD–01F D60 °C model was selected. The temperature control switch is normally closed, and when the hot surface reaches 60 °C, it can protect the semiconductor cooling plate by automatically disconnecting it and restoring the connection when the surface temperature returns to 35 °C. After connecting all the components and embedding them into the 3D–printed shell, the physical diagram of the built device is shown in Figure 13. The total weight of the device is 450 g.

### 4.2. Temperature Measurement

The overall temperature distribution of the device can be detected by an infrared thermal imaging camera. This measurement uses the IRBIS3 professional software platform, which allows for emissivity correction calibration, online monitoring of temperature trends over time within a point or area, online display of histograms, the ability to display differences in images for comparison, and tools for correction of different measurement modes. A short–focus lens was used in this test setup to take a temperature distribution map of the overall use of the portable refrigeration device as well as a temperature/time plot of the device during use.

**Figure 14:**
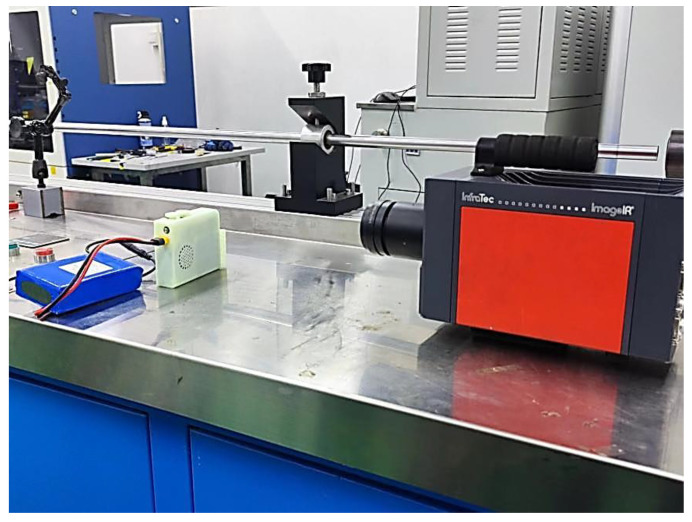
Placement of infrared thermal imager and refrigeration device.

After connecting the thermal imaging camera to the software, the “Remote Control” option is entered to select the temperature range of –20 °C to 50 °C for white balance calibration temperature. Because the principle of infrared temperature measurement uses different materials on the measurement surface of the different temperatures of infrared radiation power, the identification and processing to obtain different values of the temperature signal are different. When the received radiation power is the same, the lower the emissivity of the object surface, the greater the temperature value of the measurement result. Therefore, the emissivity needs to be calibrated before testing. The emissivity table of common materials is checked and the emissivity of white resin is obtained and input to the thermal imager. The ambient temperature is 20 °C, air humidity is 78%, and the other parameters are kept as default. The infrared thermal imager and refrigeration device are placed as shown in Figure 14.

### 4.3. Results and Discussion

**Figure 15:**
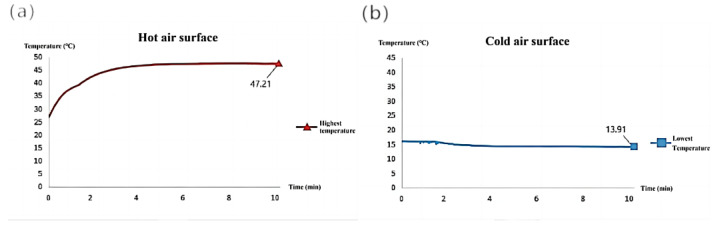
Average temperature and time curve of three measurements. (**a**) Temperature variation curve of hot air surface with time. (**b**) Temperature variation curve of cold air surface with time.

**Figure 16:**
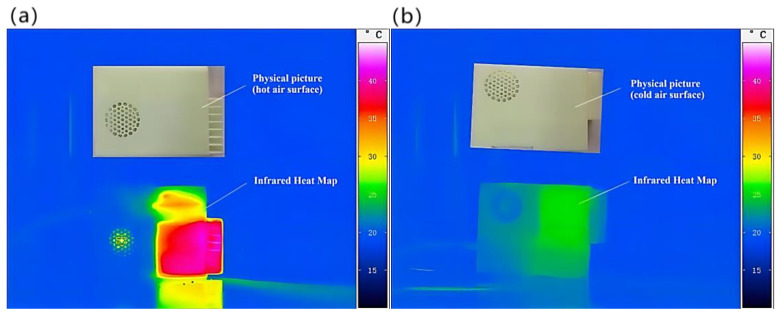
Temperature distribution results measured by infrared thermal imaging camera. (**a**) Heat map of the temperature distribution on the hot air surface of the portable refrigeration device. (**b**) Heat map of the temperature distribution on the cold air surface of the portable refrigeration device.

Boxing the measurement area of the portable refrigeration device displays in real time the temperature range and the t/s plot of the temperature within that area, i.e., the graph of the temperature over time, including the maximum, minimum, and average temperatures. When recording, the recording frequency was 2 Hz and the recording time was 10 min, and the average values of the three results are shown in Figure 15a,b. To make the imaging results more intuitive, the thermal imaging camera thermogram measurement range was adjusted in the software from 10 °C to 50 °C based on the results of the test, as shown in Figure 16a,b. For the measurement of the outlet air velocity and air volume of the portable refrigeration unit, the transmitter WD4120 was used. The wind speed, wind temperature, and air volume transmitter WD4120 is researched and designed according to the principle of hot film anemometer, with high accuracy. The test instrument used in this paper has a resolution of 0.1 m/s, is suitable for use in an environment of –40 °C to +80 °C, is powered by 24 V, and has a range of 0–10 m/s for measuring wind speed.

Through the above T/t plot as well as the thermal imaging graph, it can be concluded that the hot air surface can reach a stable state of the highest temperature of 47.2 °C in 4 min under the condition that the average temperature of the opening is about 23 °C; the cold air surface can also basically reach a stable state of the lowest temperature of 13.9 °C in 4 min under the condition that the average temperature of the opening is 23 °C. Therefore, this device only needs about 4 min to reach the stable value of cooling performance in the actual use process. The wind speed of the cold air outlet was measured at 8.24 m/s, and the wind speed of the hot air outlet was 6.92 m/s after stabilization.

Table 3 compares the simulation and test results. From the table: the portable refrigeration device based on semiconductor refrigeration technology can work normally, and its actual performance including air speed and air temperature can meet the design requirements. The cooling/heating performance and cooling/hot air outlet velocity of the device are close to the simulated results, providing reliable performance assurance for future special garments with cooling needs. The limitation of the current device is that the air volume is relatively small. The possible reasons for the difference between the simulation results and the test results are as follows: the tightness in the device is not good, and there is gas leakage, resulting in the decrease of the wind speed at the outlet. The model structure needs further optimization and improved measurement methods and instruments and so on. These possible deficiencies will be further studied in the future.

## 5. Conclusions

At present, the principles of air cooling, liquid cooling, and phase–change cooling have been applied to portable refrigeration equipment. The research shows that they can achieve certain cooling effect. However, through comparative analysis, the above technology has problems such as poor refrigeration capacity, large volume, loud noise, and so on. The principle of semiconductor refrigeration can overcome the above shortcomings, so this paper proposes and designs a new idea of semiconductor refrigeration technology applied to portable refrigeration equipment. The design scheme is constructed by three–dimensional modeling software and imported into the CFD module of ANSYS software for simulation analysis. In the simulation, the working conditions of the portable refrigeration device are set up, the model is simplified, and the performance of the device is simulated by computer. The CFD simulation results show that the device has good performance. According to the design scheme, the actual equipment is then made. The device is encapsulated in a 3D–printed shell through circuit control to realize adjustable safety protection and other related functions. The overall power consumption of the device is 82 W. An infrared thermal imaging camera and anemometer are used to measure the wind temperature and speed of the equipment. The temperature of cold air surface and hot air surface reached a stable state of 13.9 °C and 47.2 °C, respectively, within about 4 min. The experimental results are basically consistent with the simulation results, which meet the design performance requirements and verify the feasibility of miniaturization design of the device. The limitation of the current study is that the unit is a stand–alone unit and does not integrate the unit with equipment requiring cooling or heating (e.g., clothing, tents, etc.). This is also one of our future research directions. The expected idea is to attach the device to the garment so that the device can be removed and replaced easily.

## Figures and Tables

**Table 1:** Information about major components.

Tool	Manufacturer	Country	Specifications
Semiconductor cooling plate	SINHE Electronic Technology Co., Ltd.	Jiangsu, China	Size: 40 × 40 × 6.4 mm Performance: The maximum temperature difference reaches 70 °C within 40 s.
Micro–fan	Delta Electronics, Inc.	Taipei, China	Size: 50 × 50 × 10 mm Performance: Maximum air volume of 4 cubic feet per minute.

**Table 2:** Grid density independence verification results.

	Grid 1	Grid 2	Grid 3
Mesh size	0.0005 m	0.001 m	0.002 m
Maximum temperature	45.0 °C	45.0 °C	45.0 °C
Air velocity	8.6 m/s	8.6 m/s	8.6 m/s

**Table 3:** Comparison of simulation results and test results.

Position	Simulation Results	Test Results
Hot air surface	Maximum temperature 45 °C	Maximum temperature 47.2 °C
Cold air surface	Minimum temperature 8.2 °C	Minimum temperature 13.9 °C
Outlet air velocity	Hot air 8.6 m/s Cold air 8.3 m/s	Hot air 6.92 m/s Cold air 8.24 m/s

## Data Availability

Not applicable.
